# Phenotypic and Functional Profiles of Antigen-Specific CD4^+^ and CD8^+^ T Cells Associated With Infection Control in Patients With Cutaneous Leishmaniasis

**DOI:** 10.3389/fcimb.2018.00393

**Published:** 2018-11-19

**Authors:** Adriana Egui, Darién Ledesma, Elena Pérez-Antón, Andrés Montoya, Inmaculada Gómez, Sara María Robledo, Juan José Infante, Ivan Darío Vélez, Manuel C. López, M. Carmen Thomas

**Affiliations:** ^1^Molecular Biology Department, Instituto de Parasitología y Biomedicina “López Neyra”, Consejo Superior de Investigaciones Científicas, Granada, Spain; ^2^Programa de Estudio y Control de Enfermedades Tropicales, Facultad de Medicina, Universidad de Antioquia, Medellín, Colombia; ^3^Bionaturis Group, Bioorganic Research and Services, S.A., Jerez de la Frontera, Spain

**Keywords:** *Leishmania*, leishmaniasis, paraflagellar rod protein-1, biomarkers, CD8^+^ and CD4^+^ T-cells, phenotype, inhibitory receptors, Th1-cytokines

## Abstract

The host immunological response is a key factor determining the pathogenesis of cutaneous leishmaniasis. It is known that a Th1 cellular response is associated with infection control and that antigen-specific memory T cells are necessary for the development of a rapid and strong protective cellular response. The present manuscript reports the analysis of the functional and phenotypic profiles of antigen-specific CD4^+^ and CD8^+^ T cells from patients cured of cutaneous leishmaniasis (CL), patients with an active process of cutaneous leishmaniasis, asymptomatic individuals with a positive Montenegro test and healthy donors (HD). Peripheral blood mononuclear cells (PBMCs) from the patients exhibited a lymphoproliferative capacity after stimulation with total soluble protein from either *Leishmania panamensis* (S*Lp*A) or *Leishmania infantum* (S*Li*A) or with a recombinant paraflagellar rod protein-1 (rPFR1). Higher frequencies of antigen-specific T_NAIVE_ cells, mainly following stimulation with rPFR1, were observed in asymptomatic and cured patients than in patients with active cutaneous leishmaniasis, while T cells from patients with active cutaneous leishmaniasis showed a higher percentage of effector memory T cells (T_EM_ for CD4^+^ T cells and T_EMRA_ for CD8^+^ T cells). The amount of antigen-specific CD57^+^/CD8^+^ T_EMRA_ cells in patients with active cutaneous leishmaniasis was higher than that in cured patients and asymptomatic subjects. Regarding functionality, a more robust multifunctional CD8^+^ T cell response was detected in cured patients than in those with active cutaneous leishmaniasis. Moreover, cured patients showed a significant increase in the frequency of cells expressing a Th1-type cytotoxic production profile (IFN-γ^+^/granzyme-B/^+^perforin^+^). Patients with an active leishmaniosis process had a significantly higher frequency of CD8^+^ T cells expressing the inhibitory CD160 and 2B4 receptors than did cured patients. The expression profile observed in cured patients could be indicative of an imbalance toward a CD8^+^ Th1 response, which could be associated with infection control; consequently, the determination of this profile could be a useful tool for facilitating the clinical follow-up of patients with cutaneous leishmaniasis. The results also suggest a possible exhaustion process of CD8^+^ T cells associated with the evolution of *Leishmania* infection.

## Introduction

Leishmaniasis is caused by intracellular parasites belonging to *Leishmania* species, and 1.5–2 million new cases are reported annually worldwide (WHO, 2016). Therapeutic options are limited, and there is no effective vaccine (Alvar et al., 2012). Depending on the infecting *Leishmania* species, different clinical manifestations occur, with cutaneous leishmaniasis (CL) being the most prevalent clinical form (de Vries et al., 2015). In South and Central America, *Leishmania* parasites of the subgenus *Viannia* are the most prevalent etiologic agents of human CL (Castilho et al., 2010). Systematic studies carried out in different areas of Colombia since the 1980s showed the presence of six species belonging to the genus *Leishmania*, with a higher prevalence of *Leishmania panamensis* (50.8–74.5%) and *Leishmania braziliensis* (15.3–30.3%) isolates than isolates of the other species. It is thought that 97% of the pathologies caused by *Leishmania* spp. in Colombia correspond to CL (Corredor et al., 1990; Ovalle et al., 2006; Ramírez et al., 2016).

The infections caused by *Leishmania* species are, in many cases, self-healing, so it is assumed that the host immune response is a key factor that determines the pathogenesis of the infection. It has been widely reported that the Th1 response is critical for the control of *Leishmania* infection, since this response creates a cytokine environment that promotes the clearance of the parasite by macrophages (Kaye and Scott, 2011). The development of *Leishmania* infection in IFN-γ- and TNF-α-deficient murine models increased the lesion sizes and the parasite burdens (Theodos et al., 1991; Wilhelm et al., 2001; Pinheiro and Rossi-Bergmann, 2007). CD4^+^ and CD8^+^ T cells play a central role in the Th1 response by producing IFN-γ, TNF-α, and other Th1 cytokines that are essential for controlling parasite growth (da Silva Santos and Brodskyn, 2014). Thus, the cellular immune functions performed by these T cells are fundamental for eliminating the parasites, although there is evidence that CD8^+^ cytotoxic T lymphocytes (CTL) are involved in tissue damage in CL patients through cytotoxic mediators (Faria et al., 2009; Santos Cda et al., 2013). It is equally important to note that T lymphocytes play a critical role in protection against reinfection by *Leishmania* species. In this sense, after primary infection, long-lived memory T cell populations are maintained in the absence of antigens and are able to mediate immunity against a second infection (Glennie and Scott, 2016). It has been reported that cured patients who have overcome an episode of CL harbor specific effector memory T cells (T_EMs_) that produce IFN-γ and central memory T cells (T_CMs_) that produce IL-2 in response to stimulation with soluble leishmania antigens (Keshavarz Valian et al., 2013).

During the chronic stage of *Leishmania* infection, antigen-specific T cells become functionally impaired, as has been observed in other protozoan diseases (Gigley et al., 2012; Rodrigues et al., 2014). This dysfunctional process, known as T cell exhaustion, occurs gradually, with the upregulation of both the expression and coexpression of inhibitory receptor molecules in the membrane of T cells. It has been reported that CD8^+^ T cells from patients with visceral leishmaniasis exhibit an increased expression of the inhibitory receptors CTLA-4 and PD-1 (Gautam et al., 2014). In experimental models of *Leishmania donovani* infection, the blockade of the PD-1/PD-L1 pathway partially restored CD8^+^ T cell immune functions and significantly reduced the splenic parasite burden (Joshi et al., 2009; Hernández-Ruiz et al., 2010). Nevertheless, further information is needed to understand this exhaustion process in the context of *Leishmania* infection and its impact on the progression of leishmaniasis.

A systematic review of biomarkers for monitoring therapeutic responses in leishmaniasis (Kip et al., 2015) stated that sensitive and specific markers that are capable of assessing therapeutic efficacy and are able to predict long-term clinical outcomes using noninvasive sampling methods are urgently needed. The paraflagellar rod proteins (PFRs) represent a family of relevant trypanosomatid antigens that are located in the paraflagellar pocket of these parasites (Cachon et al., 1988). Knockout assays in *Leishmania mexicana* demonstrated that the proteins encoded by PFR genes play a critical role in the mobility and survival of the parasite (Santrich et al., 1997). Some members of the PFR antigen family stand out due to their high immunogenicity (Michailowsky et al., 2003). Additionally, a very recent study highlighted the potential of a recombinant PFR1 antigen for the serological diagnosis of *Leishmania infantum* infection (Ledesma et al., 2017). In the context of trypanosomatid infection by *Trypanosoma cruzi*, the immunization of mice with a protein from the PFR family (PFR2) fused to HSP70 as a DNA vaccine provided a protective response against *T. cruzi* experimental infection by inducing an increase in the expression of IL-2 and IFN-γ by splenic CD8^+^ T cells and by the generation of antigen-specific T cells (Morell et al., 2006).

In the present work, the cellular mechanisms that are triggered in human patients with different clinical statuses related to *Leishmania* infection were studied. For this purpose, a phenotypic and functional characterization of antigen-specific CD4^+^ and CD8^+^ T cells from patients cured of CL, patients with active CL (CL), asymptomatic individuals with a positive Montenegro test and healthy donors (HD) was conducted. Additionally, the degree of exhaustion and the senescence profile of these antigen-specific T cells were evaluated.

## Materials and methods

### Peripheral blood mononuclear cells isolation of the study populations

Peripheral blood was collected from Montenegro-positive subjects (*n* = 4), cutaneous leishmaniasis patients (*n* = 4), patients cured of leishmaniasis (*n* = 5), and healthy donors from endemic (*n* = 4), and nonendemic areas (*n* = 6). All individuals included in this study live in Colombia. Peripheral blood (~20 mL) from each individual was collected by venipuncture into EDTA-containing tubes (BD Vacutainer), and peripheral blood mononuclear cells (PBMCs) were purified as previously described (Egui et al., 2012). The PBMCs were suspended in inactivated fetal bovine serum (iFBS) (Gibco, Grand Island, NY) containing 10% DMSO and cryopreserved in liquid nitrogen until use.

Individuals with evidence of exposure to *Leishmania* but without clinical manifestations of disease were identified by a positive Montenegro skin test. To this end, 0.1 mL of leishmanin (provided by PECET, Antioquia University, Medellin-Colombia) was subcutaneously inoculated into the arms of healthy subjects living in a leishmaniasis-endemic region of Colombia (Caldas Department) where the most widespread species of *Leishmania* is *L. panamensis* (Corredor et al., 1990; Ovalle et al., 2006; Ramírez et al., 2016). Subjects positive to Montenegro skin test were carefully clinically evaluated. They were considered asymptomatic when it was confirmed that they never presented lesions or scarring due to *Leishmania* infection in their clinical history, and were never treated against this disease. The diagnosis of CL patients was based on combined procedures, using direct microscopic examination, parasite growth, and molecular (PCR) methods. Smear preparations from the skin lesions were fixed and stained with Giemsa for microscopic observation and also cultivated in NNN media (Robinson et al., 2002). The identification of the *Leishmania* species causing the infection was carried out by PCR-RFLP following a previously published protocol (Montalvo et al., 2008). All the collected samples corresponded to the *L. panamensis* species. *L. panamensis* infection requires specific treatment. There are no data that report spontaneous cure in patients infected with *L. panamensis* in Colombia. All the cutaneous leishmaniasis patients presented from two to six cutaneous lesions. Blood was collected from these patients before the administration of antiparasitic treatment. The enrolled patients who had been cured of leishmaniasis had not presented any cutaneous lesions compatible with leishmaniasis for at least the previous 2 years.

### Ethical considerations

The Human Research Bioethics Committee of the University of Antioquia, Medellin (reference: 16-05-727) and the Consejo Superior de Investigaciones Científicas (CSIC) in Spain (reference: 094/2016) approved the protocols used in this study. All subjects gave written informed consent in accordance with the guidelines of the Declaration of Helsinki.

### Overexpression and purification of the recombinant PFR1 protein

The cloning, expression, and purification of the *L. infantum* PFR1 antigen were performed as described previously (Ledesma et al., 2017). The overexpression of the recombinant PFR1 protein was induced by the addition of 0.2 mM isopropyl-beta-D-thiogalactopyranoside (IPTG) for 3 h at 37°C. Protein was solubilized in a buffer containing 0.3 M NaCl and 50 mM Na_2_HPO_4_ and purified by Ni^2+^-NTA affinity chromatography. The purified protein was visualized via 10% SDS-PAGE followed by Coomassie blue staining. The protein concentration was measured using a Micro BCA Protein Assay Kit (Thermo Fisher Scientific). The purified PFR1 protein was tested by an E-Toxate reaction kit (*Limulus amebocyte* lysate [LAL], Sigma), which showed that the endotoxin levels were below the detection limit of the kit (0.1 endotoxin units/mL).

### Isolation of *Leishmania panamensis* and *Leishmania infantum* total soluble antigens

*L. panamensis* and *L. infantum* promastigotes were grown in modified RPMI 1640 medium supplemented with 20% FBS and gentamycin (50 μg/mL). Promastigotes were collected by centrifugation and washed with 1 × PBS at pH 7.2. The parasites were suspended at 10^9^ parasites/mL in lysis buffer (50 mM Tris-HCl at pH 7.4, 50 mM NaCl, 0.005% NP-40, 1 mM PMSF, and 1 μg/mL leupeptin) and sonicated 3 times with pulses of 50–62 kHz for 40 s at time intervals of 20 s. The soluble protein extracts (S*Lp*A and S*Li*A) were collected by centrifugation at 10,000 rpm for 20 min at 4°C. The protein concentration was determined using the Micro BCA Protein Assay Kit (Thermo Fisher Scientific), and the proteins were visualized by 10% SDS-PAGE followed by Coomassie blue staining (Gibco).

### Cell proliferation assay

PBMCs (2 × 10^5^ cells/well) from the subjects under study were split in 96-well flat-bottom plates containing 200 μL/well RPMI-20% iFBC medium in the presence of the recombinant PFR1 antigen (5 μg/mL), S*Lp*A (10 μg/mL), S*Li*A (10 μg/mL), or 2 μg/mL concanavalin A (ConA) in triplicate. Polymyxin B (50 U/mL) was added to a parallel set of wells. The plates were incubated at 37°C in a CO_2_ atmosphere for 120 h. The cells stimulated with S*Lp*A or S*Li*A were pulsed with BrdU (10 μM) and incubated for 20 h at 37°C. Cell proliferation was determined using a nonradioactive ELISA technique, according to the manufacturer's instructions (Cell Proliferation ELISA Biotrak^TM^ System, version 2, GE Healthcare, Amersham^TM^, UK). The results were expressed as optical density (OD) values, and the stimulation index (SI) was calculated using the following formula:

$$\begin{array}{l} SI=\frac{\left[\text{OD (stimulated culture) - OD (control culture)}\right]}{\text{OD (control culture)}} \end{array}$$

The cells stimulated with rPFR1 were pulsed with methyl-^3^H] thymidine (0.5 μCi/well) and incubated for an additional 20 h at 37°C. The cells were immobilized in glass fiber filter mats using a FilterMate harvester (Perkin Elmer). ^3^H incorporation was measured in a Wallac 1450 MicroBeta counter device. The results were expressed as counts per minute (cpm), and the stimulation index (SI) was calculated using the following formula:

$$\begin{array}{l} SI=\frac{\left[\text{cpm (stimulated culture) - cpm (control culture)}\right]}{\text{cpm (control culture)}} \end{array}$$

### Monoclonal antibodies for cell surface staining

The following conjugated antibodies were included in the panel for cell surface staining: anti-CD3-Pacific Blue (clone UCHT1), anti-CD4-AlexaFluor700 (clone RPA-T4), anti-CD8-allophycocyanin-H7 (clone SK1), anti-CD27-FITC (clone M-T271), anti-CCR7-PE (clone 150503), anti-CD45RA-PerCP-Cy5.5 (clone HI100), anti-CD57-APC (clone NK-1), anti-2B4-FITC (clone 2-69), anti-TIM-3-PE-CF594 (clone 7D3), anti-CD160-AlexaFluor647 (clone BY55) (all from BD Pharmingen, San Diego, CA), and anti-PD-1-PE (clone J105; eBioscience, San Diego, CA). For the intracellular staining, the following conjugated antibodies were used: anti-CTLA-4-PE-Cy5 (clone BNI3), anti-IFN-γ-PE-Cy7 (clone B27), anti-granzyme B-PE-CF594 (clone GB11), anti-IL-2-APC (clone MQ1-17H12), anti-TNF-α-AlexaFluor488 (clone Mab11) (all from BD Pharmingen, San Diego, CA), and anti-perforin-PE (Clone B-D48; Abcam, Cambridge, U.K.). Live cells were isolated by using the Fixable Aqua Dead Cell Stain viability marker (LIVE/DEAD) (Invitrogen, Eugene, OR).

### Detection of intracellular cytokines, cytotoxic molecules, and inhibitory receptors by flow cytometric assays

PBMCs were cultured in RPMI 1640 supplemented with 2 mM L-glutamine, 10% iFBS, and 50 μg/mL gentamicin. PBMCs were cultured at 1 × 10^6^ cells/mL with anti-CD28 (1 μg/mL) and anti-CD49d (1 μg/mL) antibodies (purified clones CD28.2 and 9F10, BD Pharmingen, San Diego, CA) in the presence of S*Lp*A (1 μg/mL), in the absence of S*Lp*A (considered the basal response) or in the presence of 10 μg/mL *L. infantum* recombinant PFR1 protein (with 30 μg/mL of polymyxin B). The cells were incubated for 14 h at 37°C in a humidified atmosphere with 5% CO_2_. For intracellular staining, cells were cultured in the presence of GolgiPlug and GolgiStop according to the manufacturer's instructions (BD Pharmingen, San Diego, CA). After stimulation, the cells were washed and incubated in PBS (containing 5% iFBS) for 10 min at RT. PBMCs were stained with a viability marker, namely, LIVE/DEAD Fixable Aqua (Invitrogen, Eugene, OR), for 20 min in the dark at RT. Prior to the addition of the surface staining antibodies, the cells were washed three times with PBS (containing 5% iFBS). For the memory and phenotypic characterizations, the cells were stained with anti-CD3, anti-CD4, anti-CD8, anti-CD45RA, anti-CCR7, anti-CD27, and anti-CD57 antibodies for 20 min in the dark at 4°C. To analyze inhibitory receptor expression, the cells were stained with anti-CD3, anti-CD4, anti-CD8, anti-PD-1, anti-2B4, anti-TIM-3, and anti-CD160 antibodies for 20 min in the dark at 4°C and washed with PBS (containing 5% iFBS). Then, the cells were fixed and permeabilized with Cytofix/Cytoperm (BD Biosciences) for 20 min at 4°C, washed following the manufacturer's instructions and stained with anti-CTLA-4 for 30 min in the dark at 4°C. To evaluate the intracellular production of cytokines and cytotoxic molecules, cells were stained with anti-CD3, anti-CD4, and anti-CD8 antibodies. After washing, the cells were fixed and permeabilized with Cytofix/Cytoperm (BD Biosciences). Intracellular staining was performed with anti-granzyme B, anti-IFN-γ, anti-IL-2, anti-TNF-α, and anti-perforin antibodies for 30 min at 4°C. Finally, after the last staining step, the cells were washed, suspended in 1 × PBS and analyzed in an LSRFortessa cytometer (BD Biosciences). At least 100,000 events were acquired, and analyses were performed using FlowJo 9.3.2 (TreeStar, Ashland, OR), Pestle 1.7 and SPICE 5.3 (National Institutes of Health, Bethesda, MD). Positivity for each marker was determined using fluorescence minus one (FMO) controls. Each antibody was titrated prior to the assays. Dead and doublet cells were excluded from the analysis.

### Statistical analysis

The statistical analysis was carried out using Prism version 6.0 (GraphPad Software, La Jolla, CA). Statistical significance was calculated using nonparametric tests, such as the Mann–Whitney *U*-test (pairwise comparisons) or the Kruskal–Wallis test with the Dunn correction to identify differences between groups of cellular subpopulations or between more than two groups of subjects (ANOVA was used for comparisons among > 2 groups). Differences were considered statistically significant for *p* < 0.05, and the symbols used are ^*^p < 0.05, ^**^p < 0.01, and ^***^p < 0.001. The box plot graphs were generated by GraphPad Prism version 6.0 software and represent all values (minimum to maximum). The boxes represent the 25–75th percentiles.

## Results

### Leishmanial antigens and lymphoproliferative responses

The total soluble *Leishmania* proteins (S*Lp*A and S*Li*A) and the recombinant PFR1 protein (rPFR1) used in the assays are shown in Supplementary Figure 1. A single and intensely stained band corresponding to the PFR1 antigen with an expected molecular mass of approximately 70 kDa was observed (lane 3). The purity was higher than 95%, as determined by a band densitometry analysis following Coomassie blue staining. PBMCs from patients with active leishmaniasis showed a proliferative response after stimulation with S*Lp*A or S*Li*A, with proliferation indexes varying between 4.4 and 0.8 and between 4.3 and 0.8, respectively (Table 1). PBMCs from asymptomatic subjects with a positive Montenegro test showed proliferation indexes between 3.1 and 0.7 when stimulated with S*Lp*A and between 1.5 and 0.1 when stimulated with S*Li*A (Table 1). The proliferation indexes of PBMCs from cured patients ranged within tighter intervals, between 1.3 and 0.1 and between 0.6 and 0.02 after stimulation with S*Lp*A or S*Li*A, respectively (Table 1). A proliferative response was also detected after the stimulation of PBMCs with rPFR1. This response was stronger in cells from patients with active CL, with values ranging from 2.3 to 0.4, than in PBMCs from cured patients, with values between 0.8 and 0.02 (Table 1). Stimulation with rPFR1 did not lead to a detectable proliferative response in PBMCs from asymptomatic subjects. In general terms, and in spite of the limitation regarding to the wide range of proliferative response after antigen stimulation (S*Lp*A S*Li*A or rPFR1), the obtained results allowed to detect which group of the subject responded with a higher and lower value of the proliferative index.

**Table 1:** *In vitro* cell proliferative response to *Leishmania* antigens.

**Group of patients^a^**	**Stimulation index range**		
	**S*Lp*A**	**S*Li*A**	**rPFR1**
Asymptomatic	3.1 to 0.7	1.5 to 0.1	Nd^*^
Cured	1.3 to 0.1	0.6 to 0.02	0.8 to 0.02
Active CL	4.4 to 0.8	4.3 to 0.8	2.3 to 0.4

*The data show the proliferation index range after the stimulation of PBMCs with soluble protein extracts of L. panamensis (SLpA), L. infantum (SLiA) and the recombinant PFR1 protein (rPFR1)*.

a *Asymptomatic, asymptomatic subjects with positive Montenegro skin test. Cured, patients cured of cutaneous leishmaniasis. Active CL, patients with active cutaneous leishmaniasis*.

* *Nd, not detected*.

*All experiments were performed in triplicate*.

*The cut-off point is 0. Values above 0 in the three replicates are considered positive values. Values 0 are referred as not detected (Nd)*.

### Phenotypic characterization of antigen-specific memory CD4^+^ and CD8^+^ T cells

To evaluate the existence of a possible association between the distribution of antigen-specific memory T cells and the clinical status of CL patients, we analyzed the phenotypes of CD4^+^ and CD8^+^ T cells from PBMCs isolated from all recruited subjects except the HD (13 individuals). The expression of CD45RA, CCR7, CD27, and CD57 in PBMCs stimulated with either S*Lp*A or rPFR1 was analyzed by flow cytometry after staining with conjugated antibodies.

**Figure 1:**
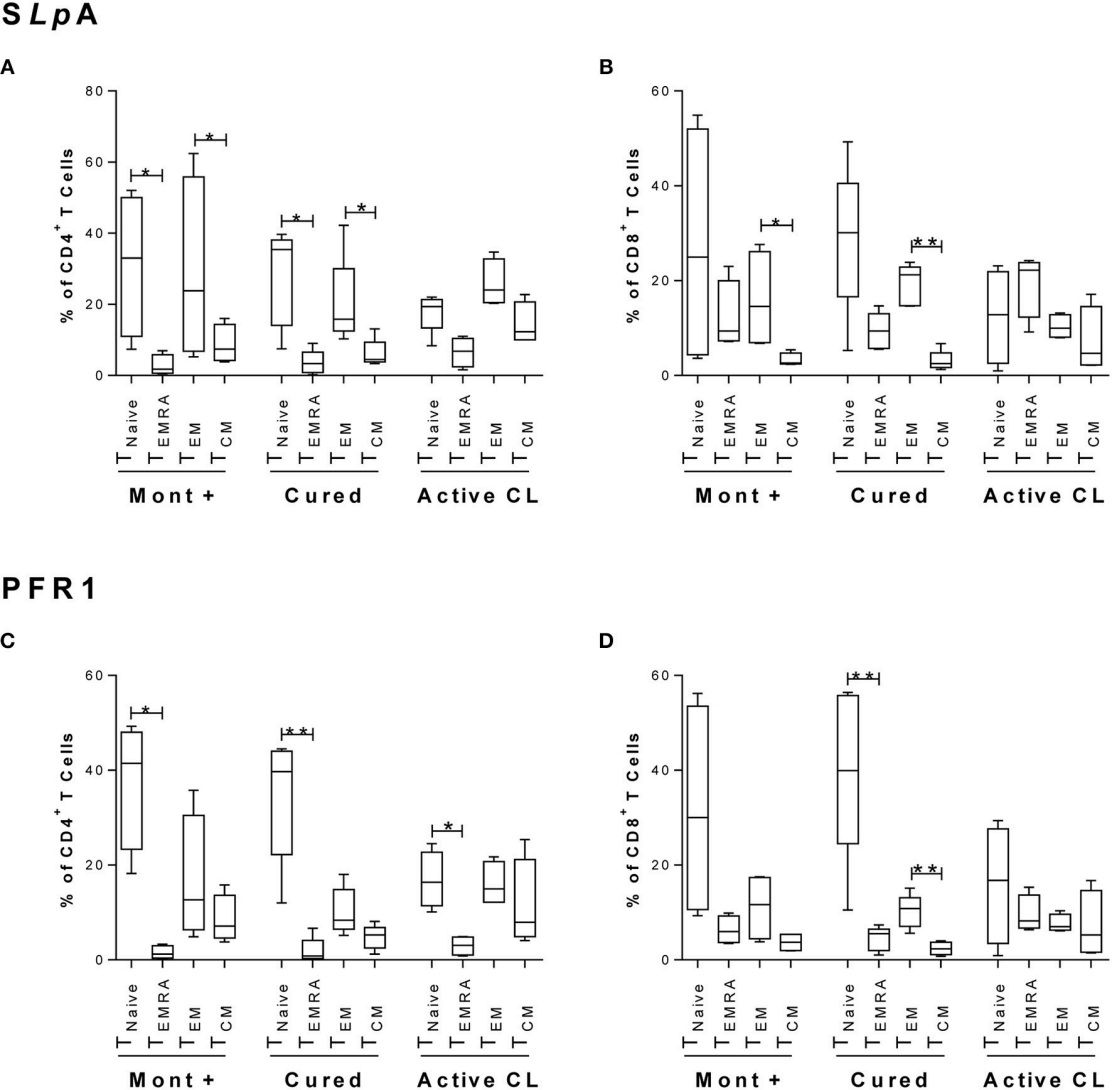
Immunophenotyping of antigen-specific CD4^+^ and CD8^+^ memory T cells. Phenotypic characterization of CD4^+^ **(A**,**C)** and CD8^+^ **(B**,**D)** T cells from asymptomatic subjects with a positive Montenegro skin test (Mont +, *n* = 4), patients cured of cutaneous leishmaniasis (Cured, *n* = 5) and patients with active cutaneous leishmaniasis (Active CL, *n* = 4). This profile was determined after stimulation with S*L*A from *L. panamensis* **(A,B)** or with the rPFR1 antigen **(C,D)**. According to CD45RA, CD27, and CCR7 expression, the cells were grouped as T_NAIVE_ (CD8^+^CD45RA^+^CCR7^+^), T_EMRA_ (CD8^+^CD45RA^+^CCR7^−^), T_EM_ (CD8^+^CD45RA^−^CCR7^−^), and T_CM_ (CD8^+^CD45RA^−^CCR7^+^). The boxes (25th−75th percentiles) and whisker plots (using the Tukey's method) show the median frequency and range of the CD4^+^ and CD8^+^ T cells. Statistical analyses were carried out using the Mann–Whitney *U*-test. Statistically significant differences were indicated by ^*^p < 0.05 and ^**^p < 0.01.

As shown in Figure 1A, within the subset of S*Lp*A-specific CD4^+^ T cells from either asymptomatic individuals with a positive Montenegro test or cured patients, the proportion of cells with a T_NAIVE_ phenotype (CD45RA^+^CD27^+^CCR7^+^) was significantly higher than that of cells with a terminal effector memory phenotype (T_EMRA_ cells, CD45RA^+^CD27^−^CCR7^−^) (*p* < 0.05). This pattern was also observed in S*Lp*A-specific CD8^+^ T cells from both groups of subjects (Figure 1B), although the difference was not statistically significant. Patients with active leishmaniasis showed a higher percentage (statistically nonsignificant) of S*Lp*A-specific CD8^+^ T cells with a T_EMRA_ phenotype than of cells with a T_NAIVE_ phenotype. Remarkably, the frequency of T_EMRA_ CD8^+^ T cells was greater in patients with active CL than in cured patients (Figure 1B). In addition, the frequency of CD4^+^ and CD8^+^ effector memory T cells (T_EM_, CD45RA^−^CCR7^−^CD27^−^) was significantly higher than the frequency of CD4^+^ and CD8^+^ central memory T cells (T_CM_, CD45RA^−^CCR7^+^CD27^+^) in both asymptomatic and cured patients (*p* < 0.05 and *p* < 0.01, respectively; Figures 1A,B).

Regarding the PFR1-specific CD4^+^ T cells, a similar phenotypic profile with a significantly higher percentage of T_NAIVE_ cells than of T_EMRA_ cells was observed in the three groups of subjects (*p* < 0.05 and *p* < 0.01; Figure 1C). Interestingly, in PFR1-specific CD8^+^ T cells, the difference between the proportions of T_NAIVE_ and T_EMRA_ cells was less pronounced in patients with active CL (Figure 1D) than in patients in the other two groups. Within this subset of CD8^+^ T cells, statistically significant differences between the proportions of T_NAIVE_ and T_EMRA_ cells were detected only in the group of cured patients (*p* < 0.01; Figure 1D). Furthermore, in this group of patients, a significantly higher frequency of CD8^+^ T cells with a T_EM_ phenotype than of CD8^+^ T cells with a T_CM_ phenotype was observed (*p* < 0.01; Figure 1D).

**Figure 2:**
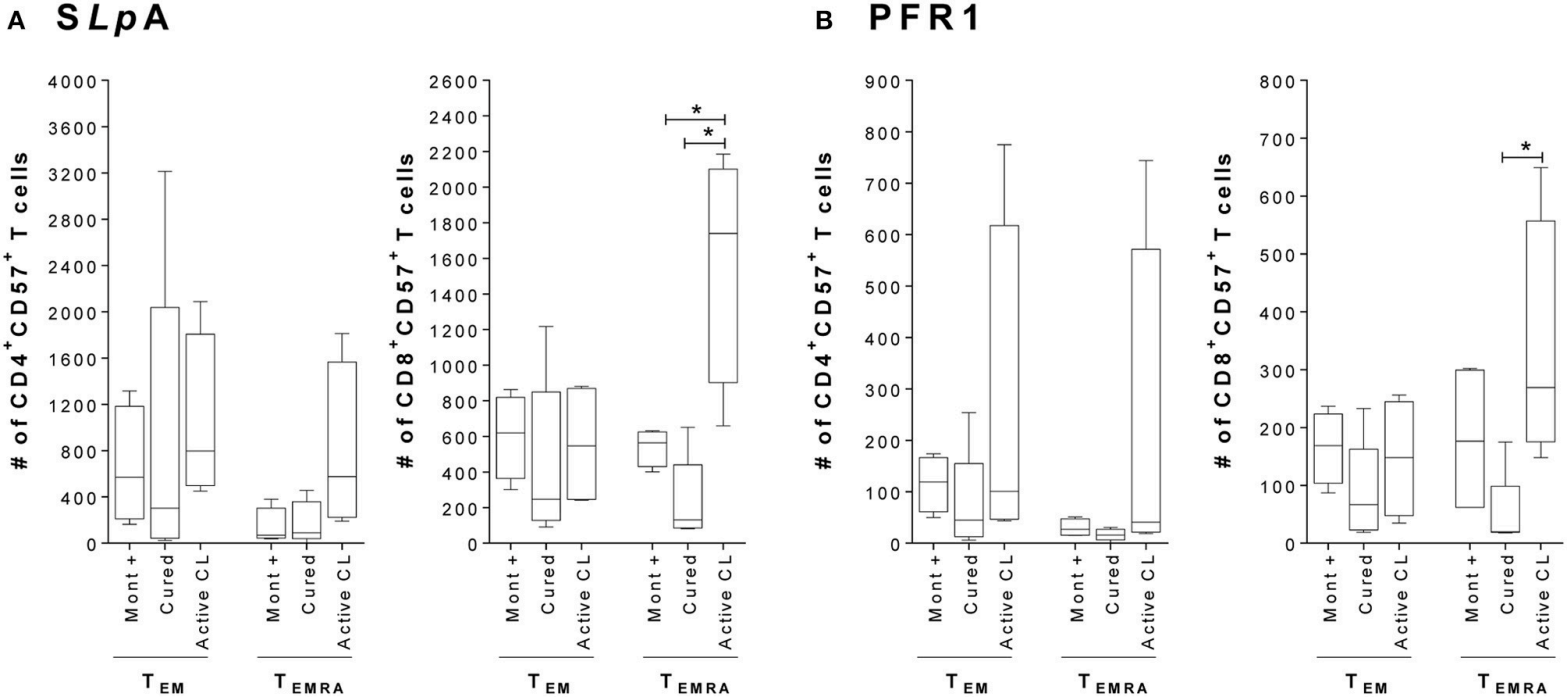
Expression of the senescence marker CD57 in CD4^+^ and CD8^+^ effector memory T cells. **(A)** Total number of S*Lp*A-specific CD4^+^ and CD8^+^ T cells that expressed the marker CD57^+^ within the T_EMRA_ and T_EM_ effector memory subpopulations. **(B)** Total number of rPFR1-specific CD4^+^ and CD8^+^ T cells that expressed the marker CD57^+^ within the T_EMRA_ and T_EM_ effector memory subpopulations. Analyses were carried out in asymptomatic subjects with a positive Montenegro skin test (Mont +, *n* = 4), patients cured of cutaneous leishmaniasis (Cured, *n* = 5) and patients with active cutaneous leishmaniasis (Active CL, *n* = 4). The boxes (25th−75th percentiles) and whisker plots (using Tukey's method) show the median and range of the CD4^+^ and CD8^+^ T cells. Statistical analyses were carried out using the Mann–Whitney *U*-test. Statistically significant differences are indicated by *p < 0.05.

Since the expression of the CD57 marker has been associated with an irreversible senescence state (replicative senescence) in cells, which is more prevalent in effector memory T cells and especially in those with a highly differentiated phenotype (Xu and Larbi, 2017), CD57 expression was evaluated in effector memory T cells (T_EM_ and T_EMRA_). The results indicate that the frequency of CD4^+^ and CD8^+^ T cells expressing CD57 tended to be higher (although not statistically significant) in T_EMRA_ cells from patients with active CL than in T_EMRA_ cells from either asymptomatic individuals with a positive Montenegro test or cured patients (Supplementary Figure 2). Remarkably, this difference was statistically significant within both subsets of S*Lp*A- and PFR1-specific CD8^+^ T cells when the evaluation was made by considering the absolute number of CD57^+^ cells within the subset of T_EMRA_ cells (*p* < 0.05; Figure 2). The CD4^+^ T cells exhibited a similar behavior, although the observed differences were not statistically significant.

### Functional capacity of circulating CD4^+^ and CD8^+^ T cells in response to *Leishmania* antigens

**Figure 3:**
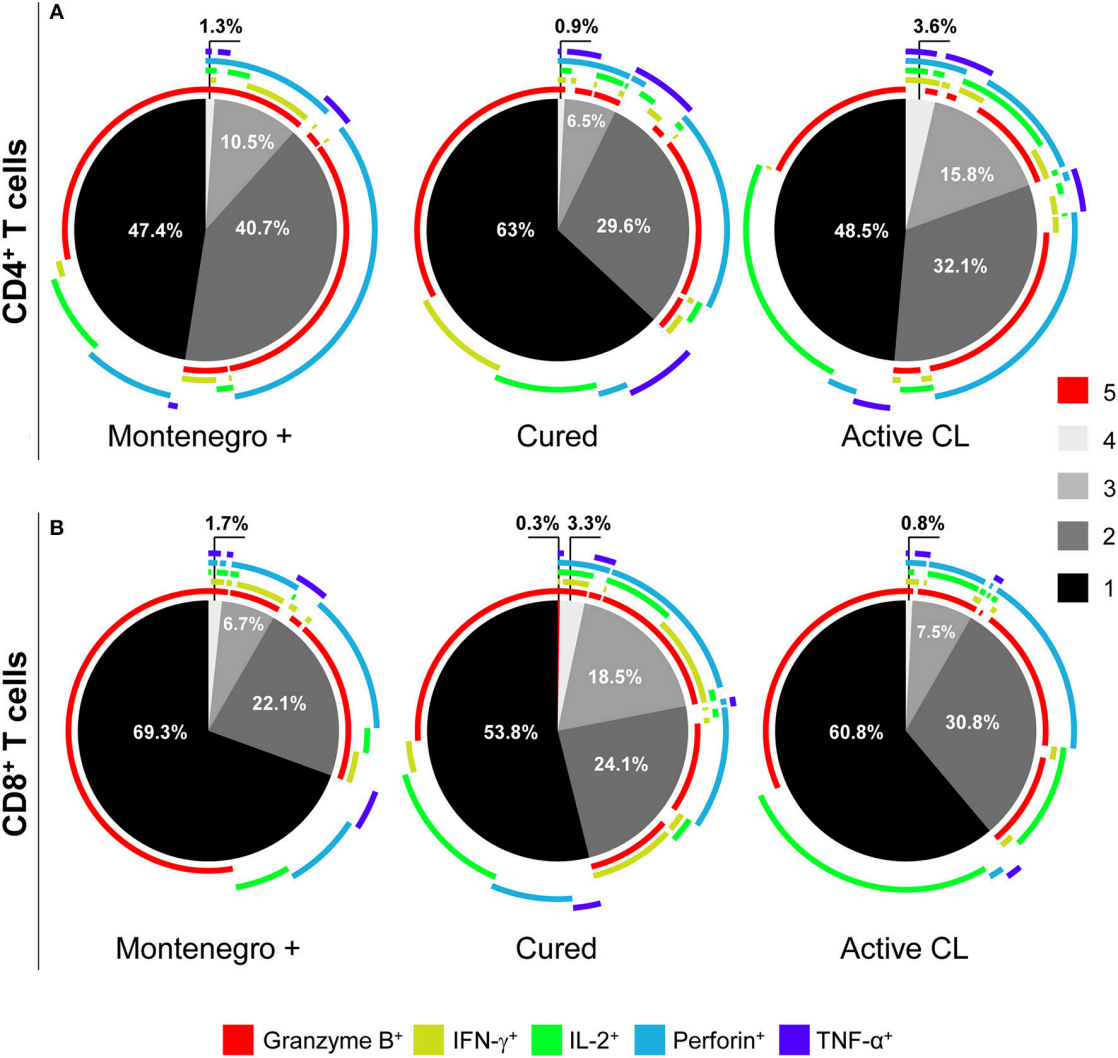
Multifunctional capacity of *Leishmania*-specific CD4^+^ and CD8^+^ T cells. Multifunctional activity of CD4^+^ **(A)** and CD8^+^ **(B)** T cells from asymptomatic subjects with a positive Montenegro skin test (Mont +, *n* = 4), patients cured of cutaneous leishmaniasis (Cured, *n* = 5) and patients with active cutaneous leishmaniasis (Active CL, *n* = 4). This profile was determined using a five-function assay to simultaneously measure the expression of IFN-γ, IL-2, TNF-α, granzyme B, and perforin after stimulation with S*L*A from *L. panamensis*. The color of each portion of the pie charts depicts the number of molecules produced by CD4^+^ and CD8^+^ T cells in response to *L. panamensis* antigens. The arcs of the pie charts represent the proportions of cells expressing each of the analyzed molecules. The percentage of unstimulated CD4^+^ and CD8^+^ T cells expressing these molecules (basal response) was subtracted from the value obtained following antigen stimulation.

To determine if there was a relationship between the pattern of functional responses and disease status, the functional responses of antigen-specific CD4^+^ and CD8^+^ T cells from asymptomatic subjects with a positive Montenegro skin test, patients cured of leishmaniasis and patients with active CL were evaluated. The production of intracellular cytokines (IL-2, IFN-γ, and TNF-α) and cytotoxic molecules (granzyme B and perforin) after stimulation with S*L*A from *L. panamensis* was determined. The results showed that the frequency of CD4^+^ T cells with multifunctional capacity tended to be greater in both asymptomatic individuals and patients with active CL than in cured patients (Figure 3), based on the greater proportions of antigen-specific CD4^+^ T cells performing four (1.3 and 3.6% vs. 0.9%, respectively) or three (10.5 and 15.8% vs. 6.5%, respectively) functions. Asymptomatic patients showed a higher frequency of bifunctional CD4^+^ T cells (40.7%) and of CD4^+^ T cells expressing both of the cytotoxic molecules, namely, granzyme B and perforin (Figure 3A). Remarkably, the evaluation of the multifunctional response in CD8^+^ T cells led to different results. The frequency of multifunctional CD8^+^ T cells was a greater although not statistically significant in cured patients than in either patients with active CL or asymptomatic patients. In fact, CD8^+^ T cells performing five functions (granzyme B^+^, IFN-γ^+^, IL-2^+^, perforin^+^, and TNF-α^+^) were detected only in the group of cured patients (Figure 3B). Similarly, higher frequencies of CD8^+^ T cells expressing four (3.3% vs. 1.7 and 0.8%) and three markers (18.5% vs. 6.7% and 7.5%) were observed in patients cured of CL than in either the asymptomatic Montenegro-positive individuals or those with active CL, respectively (Figure 3B). Furthermore, cured patients showed a greater frequency of CD8^+^ T cells expressing IFN-γ, perforin and granzyme B than did either asymptomatic Montenegro-positive individuals or patients with active CL (11.8 vs. 6.7 and 1.2%, respectively; Figure 3). In addition, while the subjects cured of leishmaniasis exhibited monofunctional cells that individually expressed all the analyzed molecules, CD8^+^ T cells from patients with active CL preferentially expressed granzyme B or IL-2 (Figure 3).

**Figure 4:**
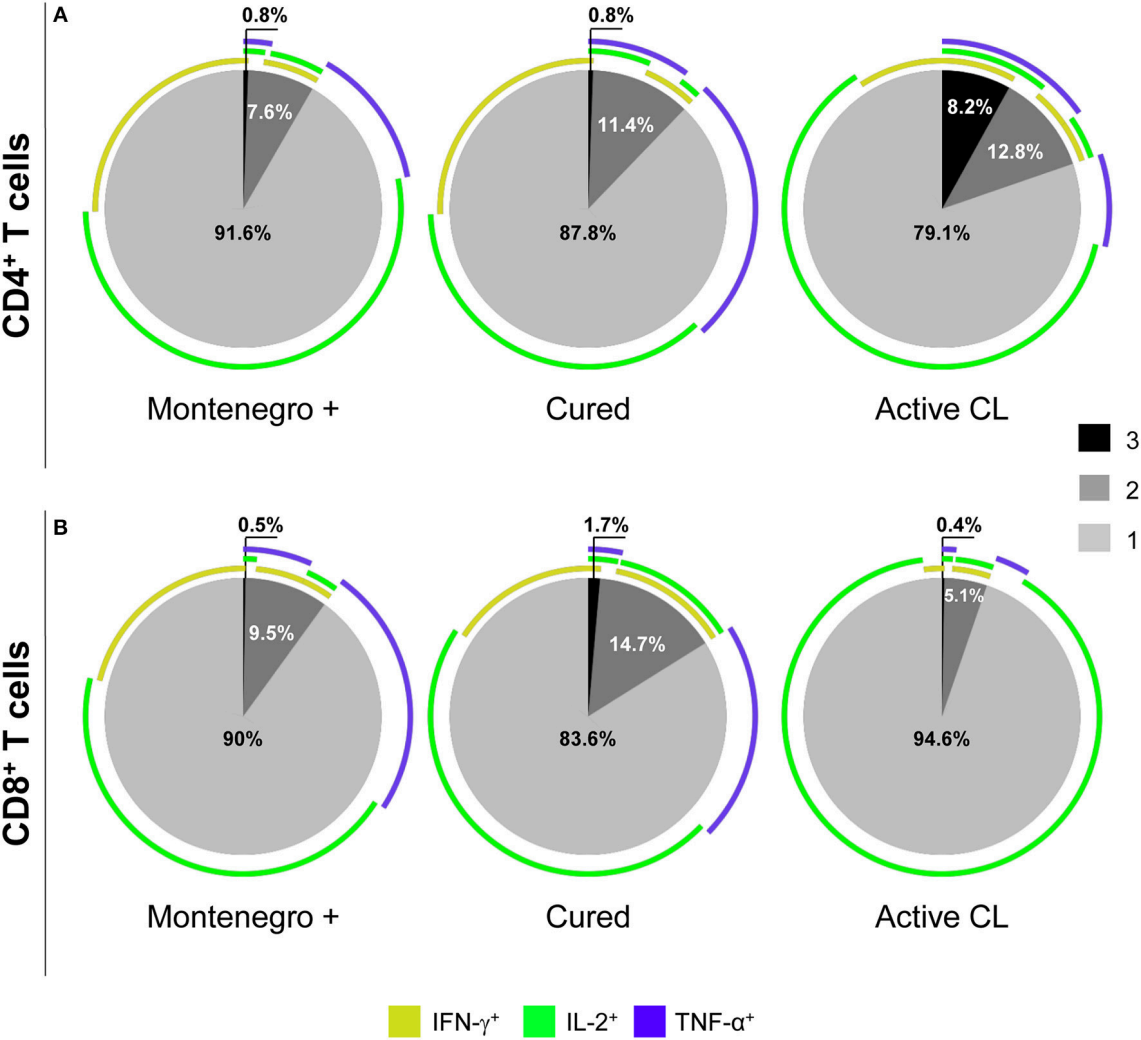
Functional capacity of *Leishmania*-specific CD4^+^ and CD8^+^ T cells. Cytokine production (IFN-γ, IL-2, and TNF-α) by CD4^+^ **(A)** and CD8^+^ **(B)** T cells from asymptomatic subjects with a positive Montenegro skin test (Mont +, *n* = 4), patients cured of cutaneous leishmaniasis (Cured, *n* = 5) and patients with active cutaneous leishmaniasis (Active CL, *n* = 4) in response to S*L*A from *L. panamensis*. The colors in the pie charts depict the presence of one, two, or three functions. The arcs of the pie charts represent the proportions of cells expressing each of the molecules under study.

**Figure 5:**
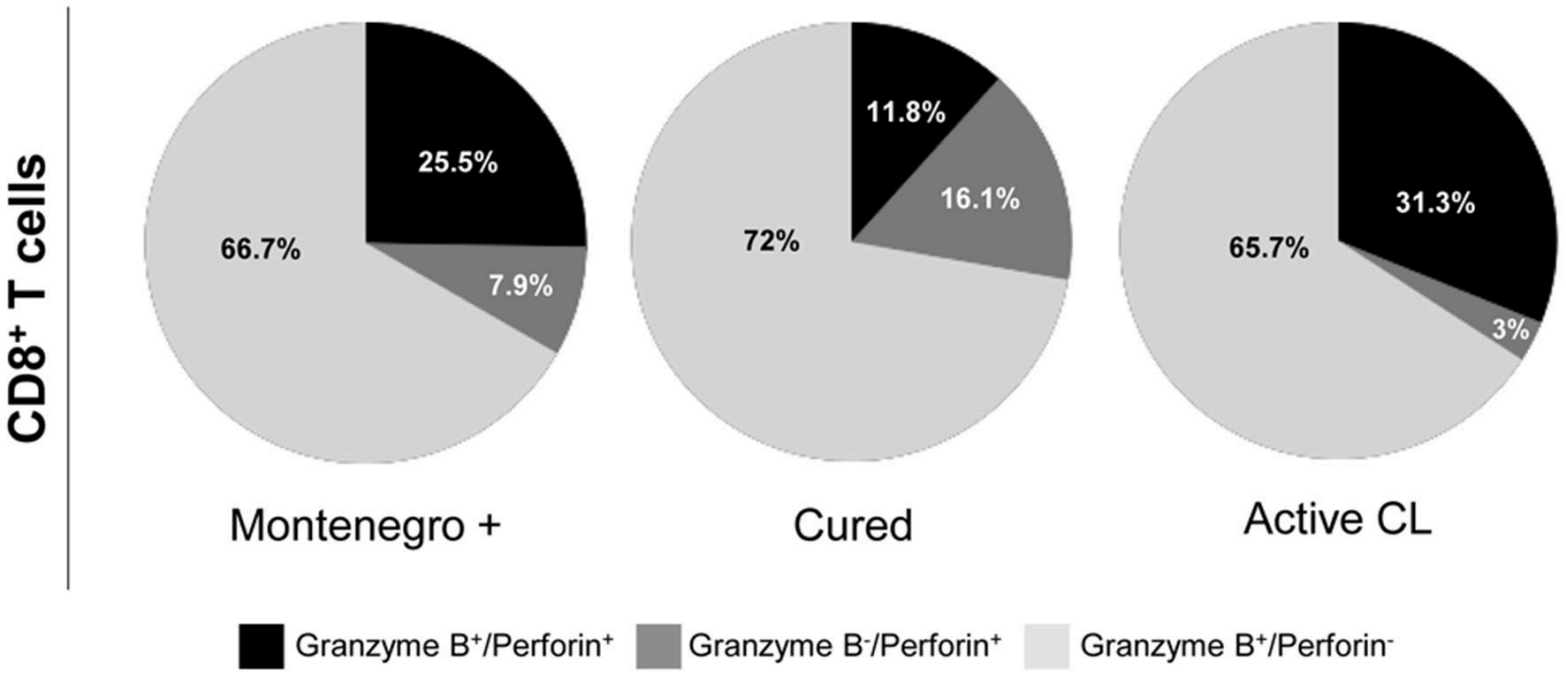
Cytotoxic profile of *Leishmania*-specific CD8^+^ T cells. Functional activity of CD8^+^ T cells determined by perforin and granzyme B expression after stimulation with S*L*A from *L. panamensis* in asymptomatic subjects with a positive Montenegro skin test (Mont +, *n* = 4), patients cured of cutaneous leishmaniasis (Cured, *n* = 5) and patients with active cutaneous leishmaniasis (Active CL, *n* = 4). The colors in the pie charts depict the proportion of cells expressing each of the analyzed molecules.

As shown in Figure 4, patients with active CL had a higher proportion of CD4^+^ T cells that simultaneously produced the cytokines IFN-γ, TNF-α, and IL-2 than did either asymptomatic individuals or cured patients (8.2 vs. 0.8%; Figure 4A). In cured patients, the monofunctional CD4^+^ T cells mainly expressed TNF-α or IFN-γ (Figure 4A), while the frequency of CD8^+^ T cells expressing three (1.7% vs. 0.5 and 0.4%) or two cytokines (14.7% vs. 9.5 and 5.1%) was higher than that in either asymptomatic individuals or patients with active CL. Conversely, patients with active CL showed a greater frequency of monofunctional CD8^+^ T cells (94.6%) than did either asymptomatic or cured patients (90 and 83.6%, respectively; Figure 4B). Our results showed that the different groups of subjects under examination might be differentiated not only based on the frequency of multifunctional CD4^+^ or CD8^+^ T cells that produce several cytokines but also based on the patterns of the expressed cytokines. For example, compared with its production in either asymptomatic individuals or cured patients, IL-2 was mainly produced by monofunctional CD4^+^ and CD8^+^ T cell populations from patients with active CL (Figures 4A,B). Moreover, the frequency of CD8^+^ T cells expressing both of the cytotoxic molecules, namely, granzyme B and perforin, was significantly lower in cured patients than in either asymptomatic subjects or patients with active CL (11.8% vs. 25.5 and 31.3%, respectively; Figure 5).

### Expression of inhibitory receptors in specific CD8^+^ T cells

**Figure 6:**
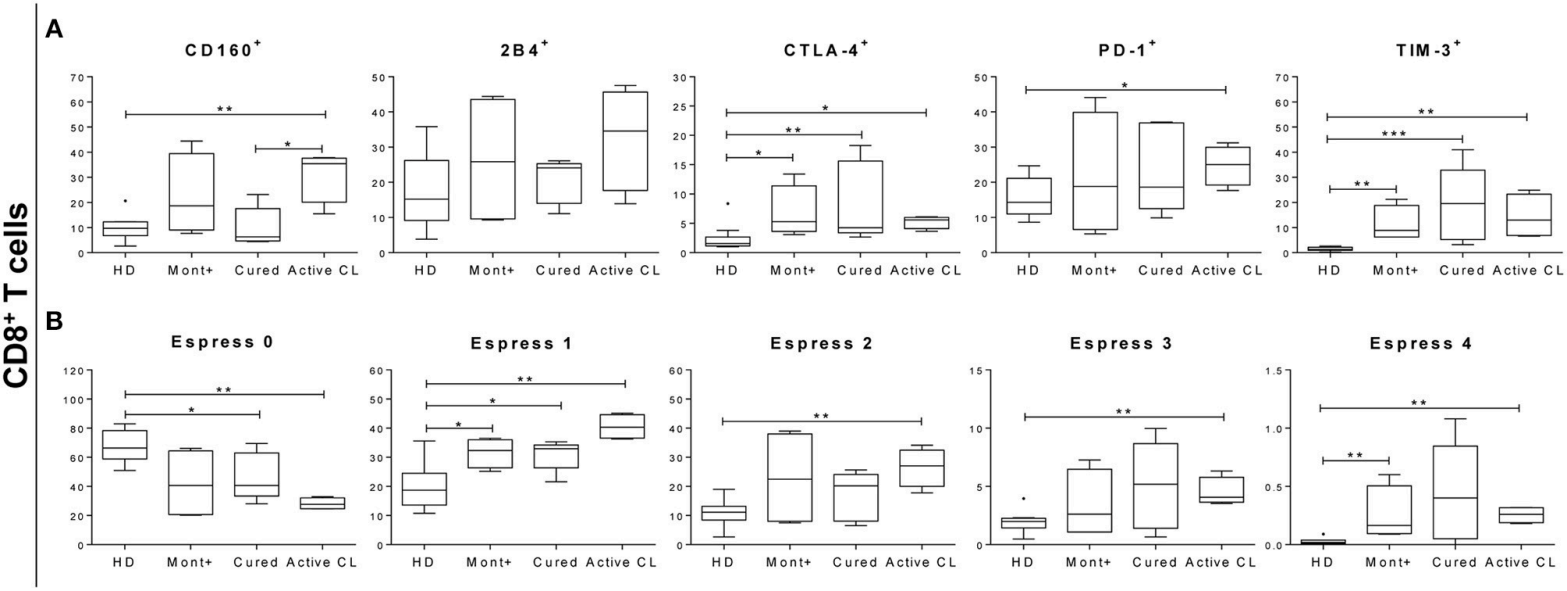
Percentage of CD8^+^ T cells expressing or coexpressing inhibitory receptors. **(A)** Frequency of CD8^+^ T cells expressing 2B4, CD160, CTLA-4, PD-1, and TIM-3 in asymptomatic subjects with a positive Montenegro skin test (Mont +, *n* = 4), patients cured of cutaneous leishmaniasis (Cured, *n* = 5), patients with active cutaneous leishmaniasis (Active CL, *n* = 4) and healthy donors (HD, *n* = 10). **(B)** Coexpression of 2B4, CD160, CTLA-4, PD-1, and TIM-3 by CD8^+^ T cells. The boxes (25th−75th percentiles) and whisker plots (using Tukey's method) show the median and range of the frequency of inhibitory receptors on T cells. Statistical analyses were carried out using the Mann–Whitney *U*-test. Statistically significant differences are indicated by *p < 0.05, **p < 0.01, and ***p < 0.001.

The next question to be evaluated was whether the functional response patterns of antigen-specific CD8^+^ T cells were associated with the expression or coexpression of inhibitory receptors. Consequently, the expression of PD-1, CTLA-4, 2B4, CD160, and TIM-3 was evaluated *ex vivo* in PBMCs from 10 HD, 4 asymptomatic subjects, 5 patients cured of cutaneous leishmaniasis and 4 patients with active CL. PBMCs were stimulated with S*Lp*A and stained using conjugated antibodies, as described in section Materials and Methods. Patients with active CL showed significantly higher frequencies of CD8^+^ T cells expressing CD160 (*p* < 0.01), CTLA-4 (*p* < 0.05), PD-1 (*p* < 0.05), and TIM-3 (*p* < 0.01) than did HD (Figure 6A). The frequency of CD8^+^ T cells expressing CD160 was also significantly higher in patients with active CL than in cured patients (*p* < 0.05). The same pattern of differences between these two pairs of patient groups was found for the frequency of CD8^+^ T cells that expressed 2B4, although the differences were not statistically significant. Further examination of the differences among samples from the different subjects revealed that the frequency of CD8^+^ T cells expressing CTLA-4 (*p* < 0.05 and *p* < 0.01) and TIM-3 (*p* < 0.01) was higher in both asymptomatic individuals and cured patients than in HD (Figure 6A). An evaluation of the concurrent expression of inhibitory receptors showed a significantly higher frequency of CD8^+^ T cells that coexpressed two, three, or four inhibitory receptors in patients with active CL than in HD (*p* < 0.01; Figure 6B). There was a higher frequency of CD8^+^ T cells that coexpressed four molecules in asymptomatic individuals than there was in HD (*p* < 0.01; Figure 6B).

## Discussion

Understanding the immunological mechanisms that underlie disease outcomes after the infection of a subject with *Leishmania (Viannia)* is essential given that there are asymptomatic patients, parasite persistence after treatment (de Oliveira Camera et al., 2006; Figueroa et al., 2009) and disease reactivation (Saravia et al., 1990). It has been reported that the clinical cure of leishmaniasis is mainly dependent on T cell populations that secrete Th1-related cytokines (Coutinho et al., 1998; Da-Cruz et al., 2005). Most knowledge obtained from the development of vaccines against CL comes from experimental infections with *Leishmania major* in murine models and cannot be fully extended to the subgenus *Viannia*. Likewise, clinical studies indicate that the human response to infection with *Leishmania (Viannia)* parasites differs from the response to infection caused by other members of the *Leishmania* subgenus (Laskay et al., 1991; Melby et al., 1994; Silveira et al., 2009). In addition, an evaluation of the treatment efficacy of several drugs against CL showed that healing is a dynamic process that involves changes in the frequency and quality of antigen-specific T cells (Da-Cruz et al., 1994; Lakhal-Naouar et al., 2015). These facts reinforce the importance of evaluating the quality of the immune response triggered by infection with *Leishmania* (*Viannia)* species (De Luca and Macedo, 2016).

In the present manuscript, the phenotype and functionality of CD4^+^ and CD8^+^ T cells from CL patients, cured patients and asymptomatic individuals with a positive Montenegro skin test were characterized. In addition, the existence of a possible CD8^+^ T cell exhaustion process associated with the evolution of *Leishmania* infection was evaluated. The results showed that cells from these patients exhibited lymphoproliferative capacity following stimulation with both S*Lp*A and S*Li*A and, to a lesser extent, with the rPFR1 protein. In active CL patients, a similar proliferative response was observed in T cells stimulated with either S*Lp*A or S*Li*A. In cured and asymptomatic patients, the greatest proliferative response was observed following cell stimulation with S*Lp*A. The cell proliferation assay with soluble antigens of *L. infantum* showed a proliferative response in all subjects, although with lower values compared to those obtained with S*Lp*A. The proliferative capacity of the subjects against the antigens of *L. infantum*, was performed as the rPFR1 protein came from *L. infantum*. These data are expected since all patients were infected with *L. panamensis*, the most prevalent infecting strain in the area where the patients under study live (Corredor et al., 1990; Ovalle et al., 2006; Ramírez et al., 2016). Consequently, subsequent cellular assays were performed using S*Lp*A as the stimulation agent.

The analysis of the phenotype of the stimulated CD4^+^ and CD8^+^ T cells from asymptomatic individuals and cured patients showed that T_NAIVE_ cells, which represented 30–40% of the total T cell population, were the subset with the highest frequency, mainly following stimulation with rPFR1. The effector memory (T_EM_) population represented ~10% of the total T cells, which was higher than the frequency of central memory T cells (T_CM_). This pattern was observed in T cells stimulated with both the soluble *L. panamensis* antigen (S*Lp*A) and the *L. infantum* rPFR1 antigen. These central and effector memory T cell profiles have been previously reported in other parasitic and viral human infections. Thus, asymptomatic and cardiac Chagas disease patients have a significantly higher proportion of T_EM_ cells than T_CM_ cells in the population of CD4^+^ and CD8^+^ T cells (Fiuza et al., 2009) both *ex vivo* and after stimulation with *T. cruzi* antigens. A similar profile was also reported in memory HIV-specific CD8^+^ T cells, for which T_EM_ cells comprised 71.8% of the population and T_CM_ cells comprised 4.1% (Champagne et al., 2001). It has been reported that after resolution of *Leishmania* infection, a small number of persistent parasites can act as source of continuous antigen stimulation to host immune cells (Sacks and Noben-Trauth, 2002), which leads to the maintenance of effector memory T cells (Aebischer et al., 1993; Mendonça et al., 2004; Okwor and Uzonna, 2008). These effector memory T cells can be maintained for prolonged durations, exhibiting increased resistance to apoptosis and playing a key role in protection against reinfection (Sallusto et al., 2004). Correspondingly, it has been reported that both central and effector memory T cells mediate long-term resistance to leishmaniasis (Zaph et al., 2004). The T cells from the studied patients with active CL showed a higher proportion of effector memory T cells (T_EM_ for CD4^+^ T cells and T_EMRA_ for CD8^+^ T cells) than T_NAIVE_ cells after stimulation with S*Lp*A. These profiles are consistent with a shift in the activation phenotype associated with the persistence of the lesions. However, the patients with active CL had similar frequencies of CD4^+^ T_NAIVE_ and T_EM_ cells after stimulation with rPFR1. The rPFR1-specific CD8^+^ T cells presented a slightly higher proportion of T_NAIVE_ cells than of T_EMRA_ or T_EM_ cells. The higher frequency of effector cells detected in patients with active CL than in either asymptomatic or cured patients could be caused by the existence of a high parasite load and the consequent activation of an antigen-specific immune response. In this context, it has been reported that chronic Chagas disease patients in the asymptomatic stage have a higher proportion of T_NAIVE_ antigen-specific CD8^+^ T cells, while chronic Chagas patients with cardiac symptomatology present a greater frequency of effector memory cells (T_EM_ and T_EMRA_) (Egui et al., 2015). The T cell-mediated immune response has also been analyzed in the skin lesions of CL patients by other authors. Thus, in CL patients infected with *Leishmania braziliensis*, a higher percentage of CD4^+^ and CD8^+^ T_EM_ cells than others T cell subsets (T_NAIVE_ and T_CM_ cells) was found at the lesion level than in the blood (de Oliveira Mendes-Aguiar et al., 2016). The higher percentage of these cells together to activated cytotoxic cells was taken as an indication of the contribution of T_EM_ to immune-mediated tissue damage. It is known that the proliferative capacity of effector T cells expressing CD57 is severely and irreversibly compromised. Interestingly, the number of antigen-specific CD8^+^ CD57^+^ T_EMRA_ cells from patients with active CL was statistically significantly higher than that observed in cured patients. Likewise, a significantly higher number of CD4^+^ T_EMRA_ cells that expressed the CD57 molecule was found in patients with active CL than in cured patients and asymptomatic subjects. These T_EMRA_ cells, which have a terminally differentiated phenotype, are very susceptible to apoptosis and produce a large amount of cytotoxic molecules (Khamesipour et al., 2012). The expression of CD57 has been strongly correlated with the simultaneous production of granzyme and perforin (Chattopadhyay et al., 2009). This phenotypic pattern is consistent with the cytotoxic profile observed in antigen-specific CD8^+^ T cells from patients with active CL. These results suggest that persistent exposure to the parasite might push the effector cells toward a terminally differentiated and senescent cell phenotype. In active leishmaniasis, the prolonged parasite presence would lead to persistent cytotoxic activity against intracellular protozoa, which could lead to a higher prevalence of senescent effector CD8^+^ T cells. The results show that CD4^+^ T cells exhibit a similar behavior, although these differences are not statistically significant. This fact may be related to the CD8^+^ T cells, as they are the cells endowed with cytotoxic activity, which is the main characteristic of CD8^+^ T cells.

CD8^+^ T cells from cured patients showed a higher multifunctional capacity than did those from patients with active CL. A multifunctional capacity of antigen-specific CD8^+^ T cells, which was only observed in cured patients, was indicated by the ability of the cells to simultaneously perform the five examined functions (IFN-γ^+^, TNF-α^+^, IL-2^+^, granzyme B^+^, and perforin^+^). In addition, cured patients showed a higher proportion of CD8^+^ T cells expressing three or four of these cytokines and cytotoxic molecules. Similarly, cured patients, in particular, showed a higher frequency of CD8^+^ T cells expressing IFN-γ^+^ and TNF-α^+^ than did patients with active CL, suggesting a relevant role of these cytokines in infection control. In murine models of leishmaniasis, CD8^+^ T cells have been shown to exert a curative role, which has been attributed to the production of Th1-related cytokines such as IFN-γ (Ruiz and Becker, 2007; Jayakumar et al., 2011). Moreover, IFN-γ-deficient mice were more susceptible to *Leishmania* infection, with larger lesions and higher parasite burdens than their wild-type counterparts (Pinheiro and Rossi-Bergmann, 2007). Additionally, it has been reported that other mediators produced by CD8^+^ T cells (such as perforin, granzymes, and chemokines) contribute to host defense. Conversely, an exacerbation of the CD8^+^ T cell immune response and the high production of cytotoxic molecules has been associated with tissue damage (Santos Cda et al., 2013). In fact, it has been reported that the CD8^+^ T cell immune response is involved in both the resolution of disease and the pathology caused by *Leishmania (Viannia)* infection in humans, highlighting the functional heterogeneity of these cells (Coutinho et al., 1998; Bangham, 2009). Interestingly, herein, it was shown that only cured patients showed a Th1-type cytotoxic profile, as indicated by the simultaneous expression of cytotoxic molecules and IFN-γ (CD8^+^ T cells, IFN-γ^+^granzyme B^+^perforin^+^). It is well-documented that resistance to leishmaniasis is related to the development and production of Th1-type proinflammatory cytokines, which leads to the activation of macrophages and to parasite killing (Sacks and Noben-Trauth, 2002; Von Stebut et al., 2003). While cytotoxic activity induces target cell death, Th1 cytokines (such as IFN-γ and TNF-α) are involved in the development of an inflammatory response that modulates the activity of macrophages and dendritic cells. However, when these pathways are not properly regulated, inflammatory disorders and tissue damage could develop (Ribeiro-de-Jesus et al., 1998; Follador et al., 2002; Arias et al., 2014). At the lesion level, there is elevated expression of granzyme B, perforin and CD107a (Novais et al., 2013; Cardoso et al., 2015), which has been positively correlated with lesion size (Santos Cda et al., 2013). Correspondingly, CD8^+^ T cells from patients with active CL presented a higher expression level of cytotoxic molecules (granzyme B^+^perforin^+^) than did those from cured patients or asymptomatic subjects.

In the context of CD4^+^ T cells, different functional profiles of the immune response were detected among the different groups of studied patients. Thus, a higher frequency of CD4^+^ T cells with multifunctional activity was detected in patients with active CL than in cured patients. However, cured patients had more monofunctional cells expressing cytokines related to the Th1 immune response, such as IFN-γ and TNF-α. These results are consistent with those of studies carried out in CL patients, which showed that after treatment, there is a reduction in both the frequency of multifunctional CD4^+^ T cells and the amount of cytokines secreted by these cells (Lakhal-Naouar et al., 2015). In fact, the depletion of effector CD4^+^ T cells in murine models did not appear to influence resistance to infection, while the depletion of CD8^+^ T effector cells reversed the protective effect, which led to a Th2 cytokine environment related to infection susceptibility (Jayakumar et al., 2011). Although little is known regarding the role of the cytotoxic activity of CD4^+^ cells in *Leishmania* infection, it has been reported that a high frequency of CD4^+^ T cell subsets producing granzyme B is found in individuals who previously had contact with *L. major* (Naouar et al., 2014). Consistently, in the present study, the production of cytotoxic molecules by CD4^+^ T cells was mainly detected in asymptomatic subjects. However, further work is needed to determine the potential roles of these cells and to understand the implications for protection and damage.

It has been reported that the healing process of CL lesions at the end of therapy is associated with an increase in the proportion of *L. braziliensis*-reactive-CD8^+^ T cells and a decline in CD4^+^-specific-reactive T cells (Da-Cruz et al., 2002). Consistent with these findings, the results of this study show a higher proportion of multifunctional *L. panamensis*-specific CD8^+^ T cells in treated cured patients than in asymptomatic subjects and active CL patients. However, a lower frequency of multifunctional CD4^+^ T cells was found in cured patients than in asymptomatic subjects and active CL patients.

In chronic diseases, the persistence of pathogen-derived antigens induces an increase in the expression of inhibitory receptors and a gradual loss of the response capacity of antigen-specific T cells. This process, known as the exhaustion process, has been mainly described in CD8^+^ T cells (Kahan et al., 2015). Very little is known about the exhaustion process in infections caused by *Leishmania* (*Viannia*) subgenus species. In the context of Chagas disease, this process has been well-documented in CD8^+^ T cells, with a higher level of dysfunction associated with a severe stage of the disease (Lasso et al., 2015). It was also recently reported that anti-*Trypanosoma cruzi* treatment partially reverses the exhaustion process in CD8^+^ T cells, thus improving antigen-specific functionality (Mateus et al., 2017). The results obtained in the present study showed higher expression and coexpression of inhibitory receptors in CD8^+^ T cells from patients with *L. panamensis* infection than in HD. Patients with active CL showed higher expression of CD160 and PD-1 than did cured patients and asymptomatic subjects. A significantly higher coexpression of inhibitory receptors by CD8^+^ T cells was only observed when patients with active CL were compared with HD. The relatively lower level of inhibitory receptor coexpression in cured patients than in patients with active CL, which was associated with the high multifunctional capacity of the CD8^+^ T cells of cured patients, suggests the critical importance of the functional response of CD8^+^ T cells in the healing process. Even the impaired or dysfunctional CD8^+^ T cell response induced by the exhaustion process and/or the senescent cell phenotype could contribute to the maintenance of an active lesion. Thus, in experimental model of CL, mice lacking PD-L1 were markedly more resistant to infection, and the lesions they developed were smaller (Liang et al., 2006). Furthermore, it has been reported in diffuse cutaneous leishmaniasis that an increase in PD-L1 expression by monocytes is a possible mechanism used by the parasites to evade the immune response (Barroso et al., 2018). In patients with visceral leishmaniasis, elevated expression of the CTLA-4 and PD-1 inhibitory receptors has been reported (Esch et al., 2013; Gautam et al., 2014; Chiku et al., 2016). Interestingly, the expression of such markers decreased in patients with *Leishmania* infection after treatment (Gautam et al., 2014). In this context, the high production of IFN-γ and TNF-α by CD8^+^ T cells and the greater proportion of multifunctional CD8^+^ T cells observed in patients cured of CL could be associated with the lower expression of PD-1 found in these patients, which might be related to a partial reversion of the exhaustion process induced by treatment. Consistent with that theory, in experimental models of visceral leishmaniasis, it has been reported that the blockade of PD-1 reduces the parasite burden, restores the T cell proliferation capacity and increases the production of IFN-γ and TNF-α (Mou et al., 2013; Chiku et al., 2016).

The findings presented in this work improve our understanding of the poorly elucidated exhaustion process that occurs in the context of *L. panamensis* infection. In addition, the data provide evidence that antigen-specific immune response markers can be correlated with the clinical status of human patients with CL caused by *L. panamensis* and might consequently be a useful tool for facilitating the clinical follow-up of patients with CL.

## Author contributions

MCT carried out the conceptualization. MCT and MCL designed the study. DL, AM, AE, EP-A, and IG obtained the samples and materials. AE, DL, and EP-A performed the experiments. AE, EP-A, DL, and MCL analyzed the results. AE and MCL performed the data visualization. AE, EP-A, MCL, and MCT discussed the results. JI, MCL, MCT, and IV acquired funding. MCL and SR supervised the study. DL, AE, and EP-A wrote the original draft. MCT, MCL, and JI wrote and edited the final version of the manuscript. All authors read and approved the final manuscript.

### Conflict of interest statement

The authors declare that the research was conducted in the absence of any commercial or financial relationships that could be construed as a potential conflict of interest.
