# Supplementary figures and images for: Phenotypic and Functional Profiles of Antigen-Specific CD4+ and CD8+ T Cells Associated With Infection Control in Patients With Cutaneous Leishmaniasis
Source: Front Cell Infect Microbiol. 2018 Nov 19;8:393. doi: 10.3389/fcimb.2018.00393 (PMC6252334; doi:10.3389/fcimb.2018.00393)

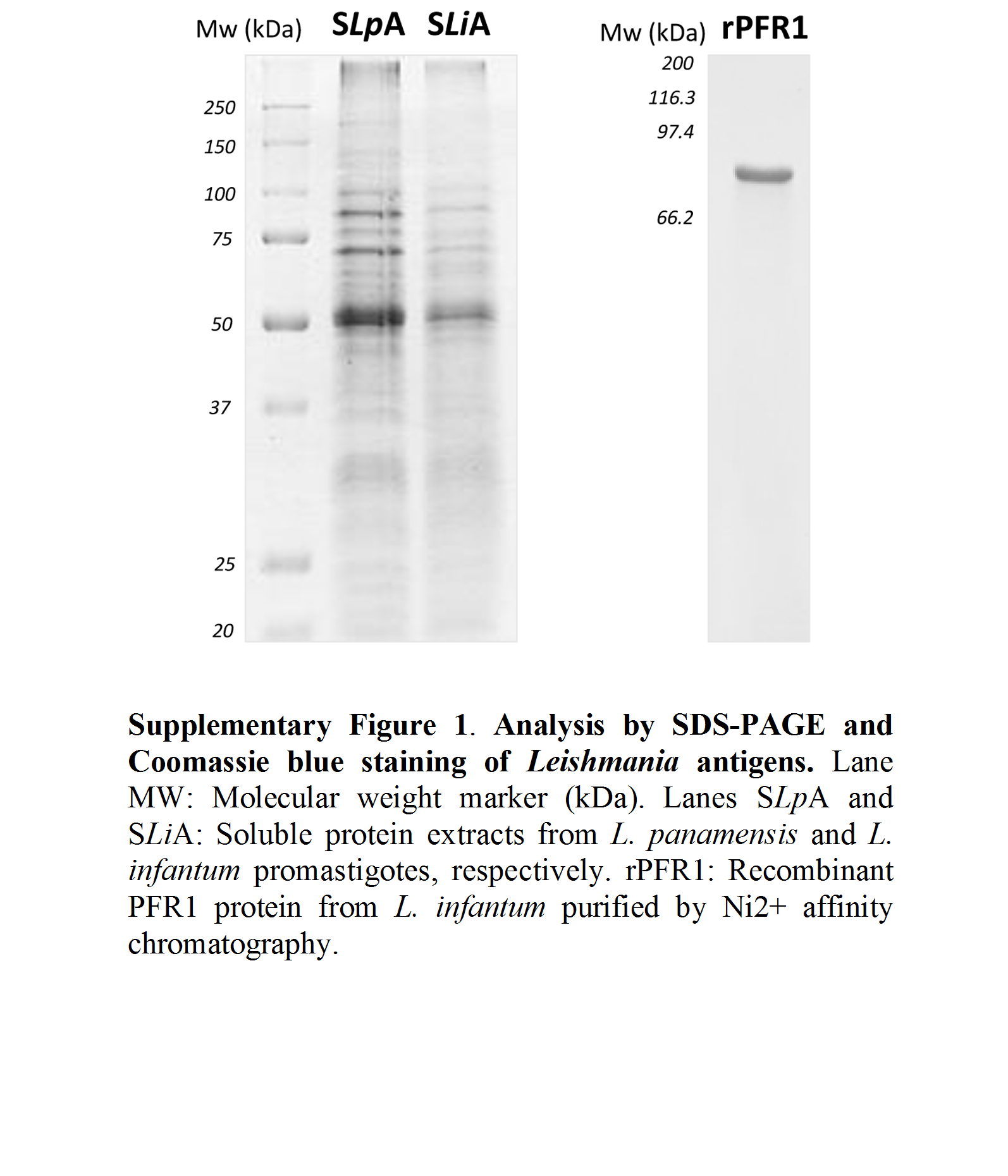

Supplement: Supplementary file 1 [file Image_1.tif]

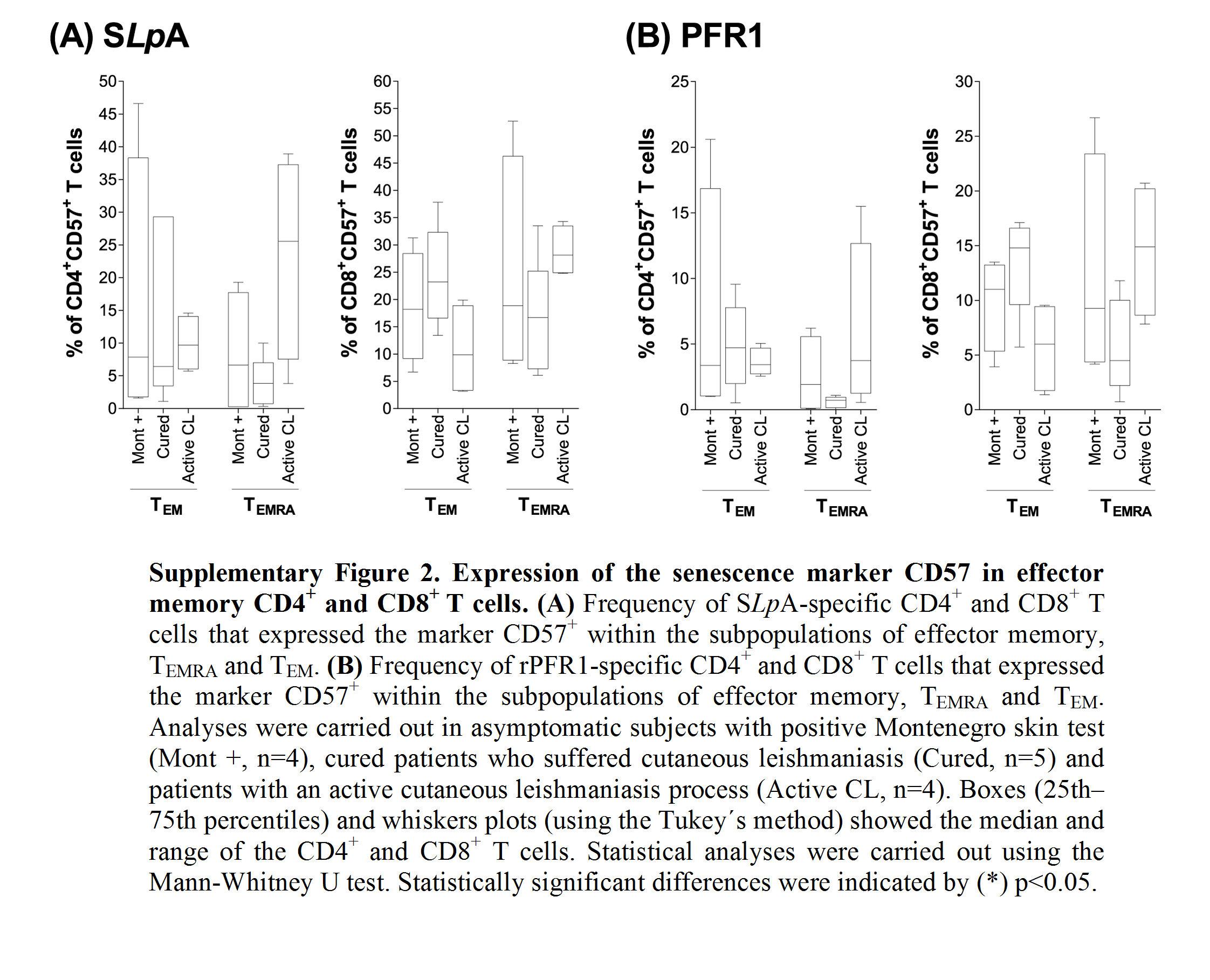

Supplement: Supplementary file 2 [file Image_2.tif]
